# Household-level and surrounding peri-domestic environmental characteristics associated with malaria vectors *Anopheles arabiensis* and *Anopheles funestus* along an urban–rural continuum in Blantyre, Malawi

**DOI:** 10.1186/s12936-018-2375-5

**Published:** 2018-06-08

**Authors:** Nicole F. Dear, Chifundo Kadangwe, Themba Mzilahowa, Andy Bauleni, Don P. Mathanga, Chifundo Duster, Edward D. Walker, Mark L. Wilson

**Affiliations:** 1 Department of Epidemiology, School of Public Health, University of Michigan, Ann Arbor, MI USA; 2 Malaria Alert Centre, College of Medicine, University of Malawi, Blantyre, Malawi; 3 College of Medicine, University of Malawi, Blantyre, Malawi; 4 Malawi International Center of Excellence for Malaria Research (ICEMR) Molecular Core Laboratory, University of Malawi College of Medicine, Blantyre, Malawi; 5 Department of Microbiology & Molecular Genetics, Michigan State University, East Lansing, MI USA

**Keywords:** Urban–rural, Urban malaria, Small-scale agriculture, Vector ecology, *Anopheles*, Malawi

## Abstract

**Background:**

Malaria is increasing in some recently urbanized areas that historically were considered lower risk. Understanding what drives urban transmission is hampered by inconsistencies in how “urban” contexts are defined. A dichotomized “urban–rural” approach, based on political boundaries may misclassify environments or fail to capture local drivers of risk. Small-scale agriculture in urban or peri-urban settings has been shown to be a major risk determinant.

**Methods:**

Household-level *Anopheles* abundance patterns in and around Malawi’s commercial capital of Blantyre (~ 1.9 M pop.) were analysed. Clusters (N = 64) of five houses each located at 2.5 km intervals along eight transects radiating out from Blantyre city centre were sampled during rainy and dry seasons of 2015 and 2016. Mosquito densities were measured inside houses using aspirators to sample resting mosquitoes, and un-baited CDC light traps to sample host seeking mosquitoes.

**Results:**

Of 38,895 mosquitoes captured, 91% were female and 87% were *Culex* spp. *Anopheles* females (N = 5058) were primarily captured in light traps (97%). *Anopheles* abundance was greater during rainy seasons. *Anopheles funestus* was more abundant than *Anopheles arabiensis,* but both were found on all transects, and had similar associations with environmental risk factors. *Anopheles funestus* and *An. arabiensis* females significantly increased with distance from the urban centre, but this trend was not consistent across all transects. Presence of small-scale agriculture was predictive of greater *Anopheles* spp. abundance, even after controlling for urbanicity, number of nets per person, number of under-5-year olds, years of education, and season.

**Conclusions:**

This study revealed how small-scale agriculture along a rural-to-urban transition was associated with *An. arabiensis* and *An. funestus* indoor abundances, and that indoor *Anopheles* density can be high within Blantyre city limits, particularly where agriculture is present. Typical rural areas with lower house density and greater distance from urban centres reflected landscapes more suitable for *Anopheles* reproduction and house invasion. However, similar characteristics and elevated *Anopheles* abundances were also found around some houses within the city limits. Thus, dichotomous designations of “urban” or “rural” can obscure important heterogeneity in the landscape of *Plasmodium* transmission, suggesting the need for more nuanced assessment of urban malaria risk and prevention efforts.

**Electronic supplementary material:**

The online version of this article (10.1186/s12936-018-2375-5) contains supplementary material, which is available to authorized users.

## Background

Malaria continues to take the lives of nearly half a million people every year, with 90% of deaths occurring in sub-Saharan Africa (SSA). The World Health Organization (WHO) estimates 216 million cases and 445,000 deaths due to malaria in 2016, an increase of approximately 5 million cases during 2015 [1]. Malaria is endemic throughout most of SSA and is the leading cause of death in Malawi among children under five years of age [2].

In 2017, approximately 3.2 million people in Malawi (17% of the population) lived in an urban setting [3, 4]. With an annual urban growth rate of 4%, Malawi has one of the highest rates of urbanization of any African country [3, 4]. Although malaria in SSA has been widely studied, most research has been carried out in rural contexts, and little is known about how increasing urbanicity may be affecting *Plasmodium* transmission and malaria risk.

Considerable evidence suggests that people living in urban settings in SSA have improved health, including decreased infant mortality, better nutritional status, increased vaccine coverage, and increased access to care [5]. Specific to malaria, studies have shown that long-lasting insecticidal net (LLIN) use is higher in urban compared to rural settings, and overall parasite prevalence in children living in large cities in SSA is less than half that of children living in rural communities within the same zone of malaria endemicity [6–8]. Urban areas generally experience lower incidence of malaria compared to rural settings, as greater human population densities may reduce individual-level exposure [9–11, 13, 14]. Less vegetation and polluted water sources may reduce the number of suitable breeding sites for *Anopheles* mosquito vectors and limit opportunities for vector dispersal from breeding sites [10–15].

Nonetheless, knowledge about local *Plasmodium* transmission in highly heterogeneous urban areas remains limited. Conditions of urban poverty, poor quality infrastructure, and small-scale crop production may enhance anopheline breeding habitats, particularly for adaptable species such as *Anopheles funestus* [12, 14, 16, 18, 19]. In addition, urban land use is often poorly monitored, especially in areas regarded as peri-urban “sprawl” [14]. Urban small-scale agriculture gardens may also provide more suitable breeding and resting sites for *Anopheles* mosquitoes, thereby contributing to local, urban *Plasmodium* transmission [17–19].

Definitions of “urban” vary widely among countries, have changed over time, and are often incomplete or not useful for studying disease [20, 21]. Traditionally, a dichotomized approach has been used to differentiate “rural” from “urban”. In developing countries where urbanization is highly variable, this approach may misclassify or fail to capture the fine-scale heterogeneity within these broad classifications, which is essential to understanding underlying drivers of various disease-causing processes [22, 23]. Understanding specific urban or rural factors that are protective against or risky for malaria should improve disease surveillance and help target control efforts [24].

This aim of this study was to characterize the diversity and abundance of *Anopheles* species along an urban–rural continuum in Blantyre, Malawi, and to assess which household-level and surrounding peri-domestic environmental characteristics are associated with increased vector abundance.

## Methods

### Study design

**Fig. 1:**
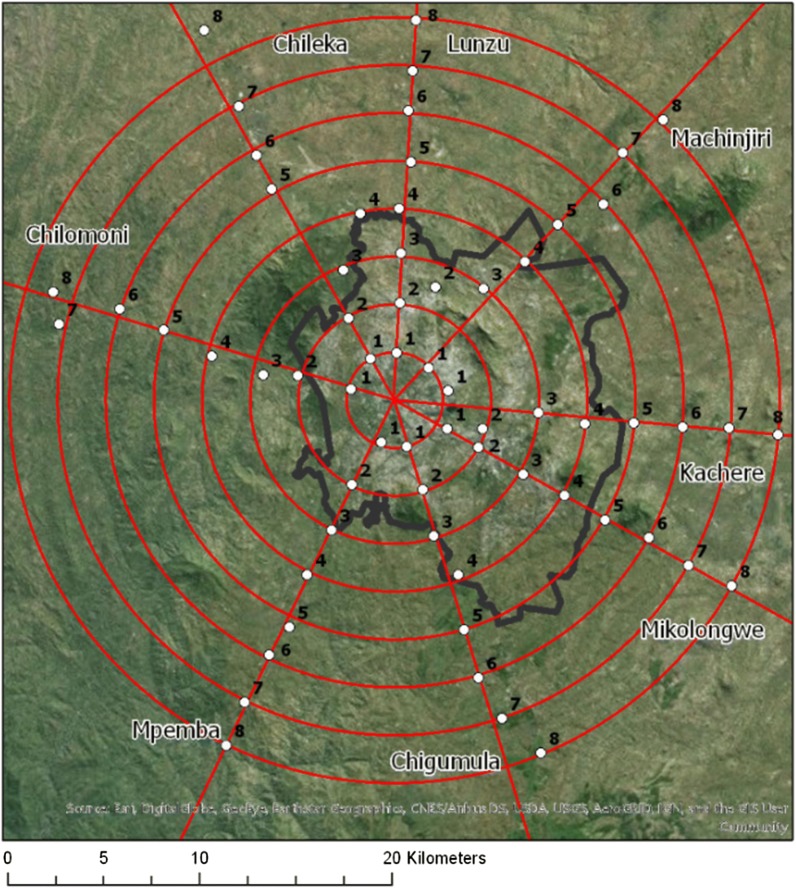
Sampling design (black boundary denotes Blantyre city administrative boundary). The eight transects were aligned with major roads leading outwards from Blantyre city centre towards rural Blantyre. Clusters of five households each within a distance of 1.5 km of the road were chosen at random from within a 500 m × 500 m area at each of the 64 sampling points. Each 500 m × 500 m area was divided into a grid of 25 subunits, each 100 m × 100 m, and five houses were chosen at random from five of the 25 subunits. If more than one household was in a 100 m × 100 m subunit, only one was selected. If fewer than five houses were located within the total 500 m × 500 m area, houses nearest to the grid were selected progressively until five households total were identified [25]

A total of 320 houses were identified for study, comprised of five houses at each of eight locations situated 2.5 km apart along each of eight transects radiating out from Blantyre city centre (Fig. 1). Houses were sampled during five, 6-week periods in both rainy and dry seasons between February 2015 and August 2016, for a possible total of 1600 house-samples. The study protocol was approved by the University of Malawi College of Medicine Research and Ethics Committee, as well as the Institutional Review Boards at Michigan State University and the University of Michigan [25].

Informed consent to administer a questionnaire and collect mosquitoes was obtained from the head of household or another adult resident of the household at the time of the first survey visit. Socio-demographic and environmental data were collected for each household and its surrounding area. Household questionnaires were administered by trained surveyors to obtain demographic and malaria risk or prevention information. Data on housing construction and peri-domestic land use/land-cover (LULC) were collected by direct observation of the house, and within a ~ 50 m radius surrounding the dwelling. House construction variables included windows (open or partially open vs. closed), eaves (open or partially open vs. closed) and roofing material (iron sheets, thatch, and tile). The presence or absence of various LULC types including any type of agriculture (maize, millet, cassava, tomato, potato, green peas and/or cocoa), fruit trees, forest, and grazing land were documented. All questionnaire and observational data were recorded on tablets using OpenDataKit collect software.

### Mosquito collection

To measure malaria vector species abundance and distribution, indoor resting adult mosquitoes were sampled using Prokopack™ aspirators, and foraging adult mosquitoes were collected using CDC miniature light traps [26, 27]. During each household visit, survey team members spent ~ 10 min. aspirating walls and ceilings of sleeping and living spaces, beneath furniture, behind curtains, and around clothing. Light traps without chemical attractants were turned on at dusk by a household member and were removed the following morning by a study team member. All mosquitoes collected by light trap or aspiration were returned the same day to the entomology lab for morphological species identification, sexing, and determination of blood-feeding by microscopy [28]. Species identification of all *Anopheles* females was later confirmed by Polymerase Chain Reaction (PCR) at the International Center of Excellence for Malaria Research (ICEMR) Molecular Core facilities at Malawi College of Medicine. Details of laboratory methods for mosquito speciation are presented in Additional file 1.

### Satellite-derived variables

Global positioning system (GPS) coordinates for each sampled household were recorded on tablets with a mean accuracy of ± 4.9 m. Multiple GPS-derived locations at the same house were averaged. Composite Google Earth imagery was extracted and analysed with ArcMap 10.2.1 (ESRI, Redlands, CA); dates ranged from January 2015 to December 2016 depending on the highest resolution image with minimal cloud cover available for each region. All households in a 50 m buffer around each observation were digitized and density was computed. Study sites were classified as “within Blantyre city limits” (urban) or “outside Blantyre city limits” (rural) in ArcMap 10.2.1 based on official governmental administrative boundary limits. Publicly available, spatially referenced data were downloaded and values at the household-level were extracted in ArcMap 10.2.1 or QGIS 2.18 (Open Source) for elevation [digital elevation model (DEM)], normalized difference vegetation index (NDVI), and percentage of cropland within a 50 m radius around each dwelling [29–31]. NDVI was calculated in QGIS 2.18 using bands 4 (Red) and 5 (near infrared (NIR)) from Landsat 8 OLI/TIRS C1 Level-1 30 m resolution satellite imagery for two time points, March 21, 2016, and July 27, 2016, corresponding to one rainy and one dry season within the study period respectively [32–34]. NDVI ranges from − 1 to + 1.

$$NDVI = \frac{NIR - Red}{NIR + Red}$$

### Statistical analyses

Descriptive statistics were summarized for all characteristics of households with non-missing exposure, outcome, and covariate data. Mosquito abundance data were summarized for rainy and dry seasons. All statistical analyses were conducted in SAS 9.4 (SAS Institute, Cary, NC).

Negative binomial regression models were used to evaluate possible associations of explanatory variables with counts of *Anopheles funestus* and *Anopheles arabiensis,* separately. Explanatory variables included those involving household demographics (number of rooms per house, number of household members, number of children under 5 years old, education status, and sex of household head), and anti-malaria behaviours (bed net ownership and use). In addition, associations between *Anopheles* abundances and household environmental and peri-domestic characteristics were evaluated, including season, elevation, urban or rural status, distance from city centre, surrounding house density, NDVI, presence of various LULC types (e.g. agriculture, forest, and grazing), livestock ownership, windows, and eaves (open or partially open vs. closed), and roof type.

Abundances of *An. funestus* and *An. arabiensis* were analysed as count data with an equal observation time of one trap-night considered for each household. Counts of *Anopheles* spp. (arabiensis and funestus) mosquitoes caught by light trap exhibited significant over-dispersion. Zero *An. arabiensis* and *An. funestus* mosquitoes were captured in 85.2 and 79.7% of households, respectively, one mosquito was caught in 5.6 and 7.1% of households, respectively, and two or more mosquitoes were caught in 9.2 and 13.2% of households, respectively.

Variances of mosquito counts exceeded means, thus Poisson and negative binomial models were compared to determine best fit. Zero-inflated models were not considered despite overdispersion of zero mosquito counts, because zero-inflated models assume that the zero outcome is due to two different processes, one process with zero being the only possible outcome. Since exposure to malaria vectors is generally ubiquitous in this study area, other types of statistical models were compared. Both Poisson and negative binomial model types gave equivalent effect estimates, but negative binomial models for both *An. funestus* and *An. arabiensis* fitted the data better based on Akaike information criterion (AIC) and the likelihood ratio test. Logistic regression models were also explored using presence or absence of *An. funestus* and *An. arabiensis* separately as the outcome variable. Observations were assumed to be independent due to the cross-sectional nature of the sampling design.

## Results

### Descriptive statistics

A total of 1548 household surveys were completed during five sample periods in 2015 and 2016. During these surveys, 1472 successful light-trap-nights captured a total of 38,895 mosquitoes (Table 1). Because aspiration capture was inconsistent and produced few adult *Anopheles* mosquitoes (of 7246 total aspiration captures, only 217 were *Anopheles* and the remainder *Culex*), no further analyses of these data were undertaken. Most mosquitoes (87%) collected by light trap were *Culex* spp., of which the majority (90%) were female *Culex.* For this report, no analyses of *Culex* mosquitoes were done as *Culex* do not contribute to the transmission of human malaria. A total of 4935 *Anopheles* spp. mosquitoes were collected using CDC light traps; female *Anopheles* made up 99% of the *Anopheles* captured (N = 4888) (Table 1).

**Table 1:** Summary of mosquitoes collected by CDC light traps

Sex	Genus	Trap nights	Total	Average per light-trap-night	Standard deviation
Female	*Culex*	1472	30,237	20.5	47.1
	*Anopheles*	1472	4888	3.3	17.6
	*Aedes*	1472	156	0.1	0.9
Male	*Culex*	1472	3526	2.4	9.7
	*Anopheles*	1472	47	0.0	0.3
	*Aedes*	1472	41	0.0	0.3

**Fig. 2:**
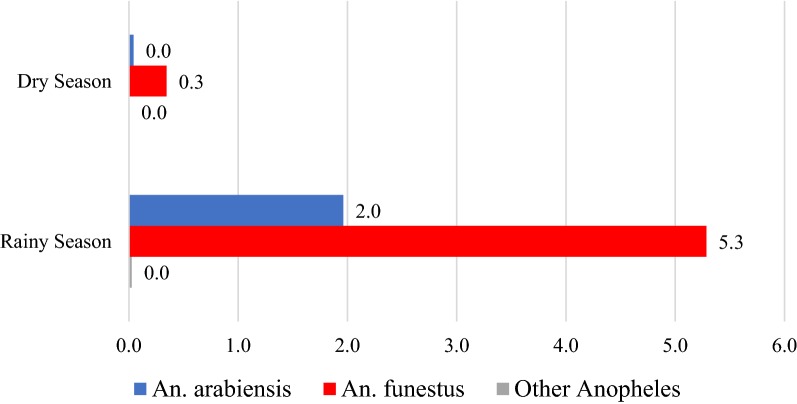
Average number of female *An. arabiensis* and *An. funestus* per light-trap-night, by season

**Fig. 3:**
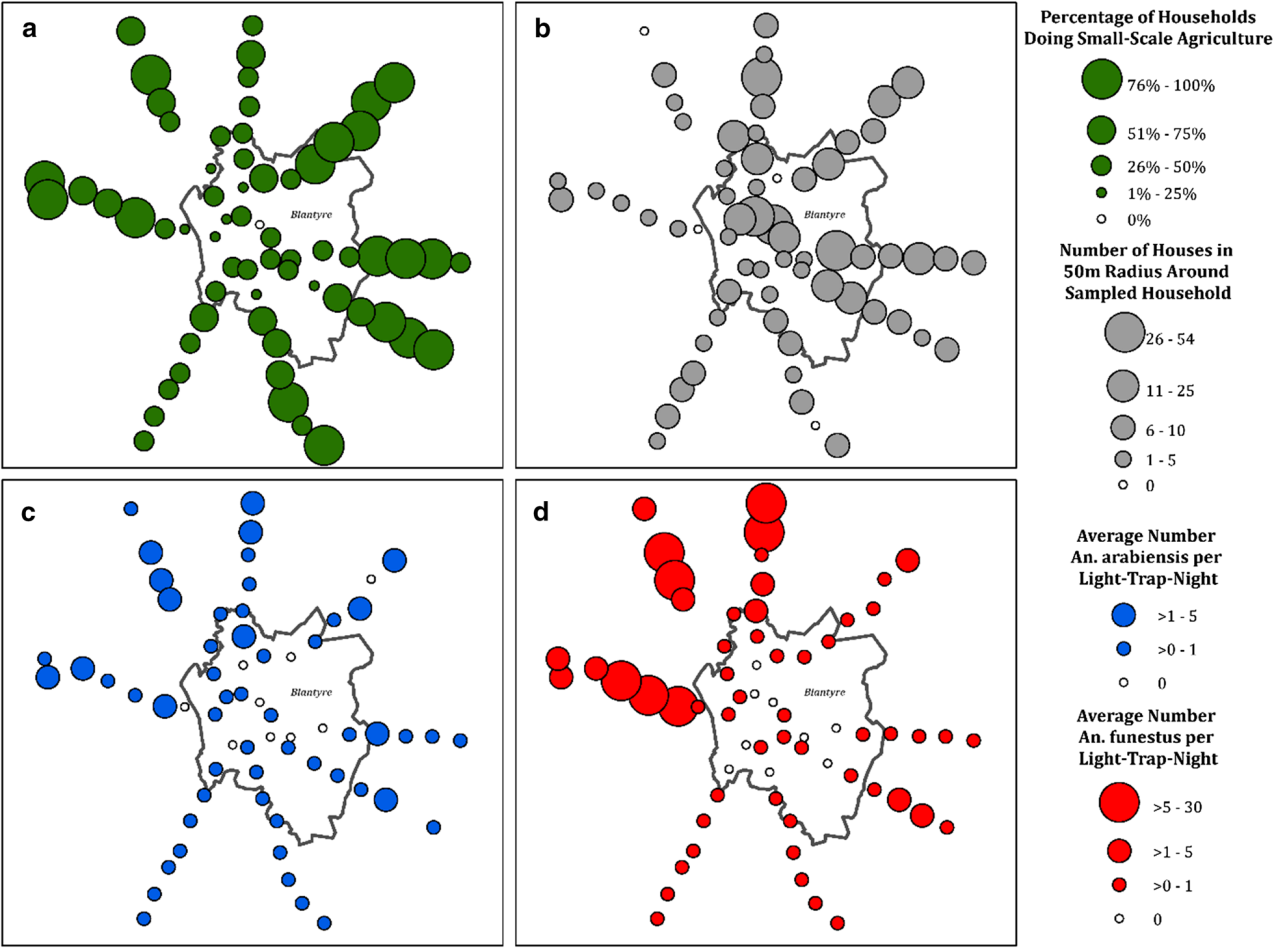
Distribution of **a** peri-domestic agriculture, **b** urban house density, **c** Female *An. arabiensis,* and **d** Female *An. funestus* for household clusters along an urban–rural continuum in Blantyre, Malawi

After identification of sex and genus by microscopy, a total of 4550 *Anopheles* spp. mosquitoes were tested by PCR to determine sibling species; *An. arabiensis* and *An. funestus* species were identified. Both *An. arabiensis* and *An. funestus* species were captured in the rainy and dry seasons with female *An. funestus* being much more abundant overall. During the rainy season, the average number of female *An. funestus* was 5.3 per light-trap-night compared to an average of 2.0 female *An. arabiensis.* During the dry season, an average of 0.3 female *An. funestus* were captured per light-trap-night compared to an average of 0 female *An. arabiensis* (Fig. 2, Table 2). While *An. funestus* was more abundant than *An. arabiensis*, both species were found on all transects, including within Blantyre city limits (Fig. 3c, d).

**Table 2:** Summary of *Anopheles* spp. mosquitoes collected by CDC light traps

Sex	Species	Trap nights	Total	Average per light-trap-night	Standard deviation
Dry season					
Female	*Anopheles arabiensis*	845	36	0.0	0.4
	*Anopheles funestus*	845	291	0.3	2.1
	Other *Anopheles*	845	2	0.0	0.1
Male	*Anopheles arabiensis*	861	0	0.0	0.0
	*Anopheles funestus*	861	4	0.0	0.1
	Other *Anopheles*	861	0	0.0	0.0
Rainy season					
Female	*Anopheles arabiensis*	578	1134	2.0	6.5
	*Anopheles funestus*	578	3055	5.3	22.0
	Other *Anopheles*	578	15	0.0	0.4
Male	*Anopheles arabiensis*	611	4	0.0	0.1
	*Anopheles funestus*	611	9	0.0	0.2
	Other *Anopheles*	611	0	0.0	0.0

Household-level data were summarized for all 1548 households with non-missing data, and included demographic characteristics, malaria risk or prevention information, and environmental data (including peri-domestic LULC and housing structure characteristics) (Table 4). Almost two-thirds (65.6%) of study households were located within “rural” Blantyre. Households had an average of 3.6 rooms, 0.6 children under 5 years of age, and a head of the household of age 40.5 years on average. About half (52.5%) of all household heads had at least some primary education, and another third (32.3%) had some secondary education. Households owned an average of 1.6 total nets, with less than one net (0.5 nets) per person on average. Nearly two-thirds (63.7%) of respondents reported that they had slept under a bed net the night prior to the survey, and 59.2% reported that other family members had slept under a bed net the night prior to the survey (Table 4).

Households were located, on average, at 945 m above sea level, with those in urban Blantyre averaging higher elevation (1057 m) compared to rural Blantyre (886 m). Forty percent (39.9%) of household-samples occurred during the rainy season and 60.1% during the dry season. Slightly over half (53.0%) of all households were growing crops at the time of sampling (direct observation), and three quarters (73.3%) had cultivated fruit trees nearby. Approximately one-fifth (17.8%) of households were raising animals for nearby grazing, with 14.3 and 37.6% reporting ownership of goats and chickens respectively. Just 7.0% of households were located near forested areas. Most houses (88.5%) had open or partially open windows, and 90.5% had open or partially open eaves. Approximately three-quarters (74.7%) of roofs were constructed with iron sheets and most of the remainder were thatch (25.1%), with < 1% tile (Table 4).

House density within 50 m of sampled households averaged 8.3 other dwellings (satellite-derived). Households dedicated an average of 25.8% of surrounding land (within a 50 m radius) to crop production (satellite-derived). The average NDVI during the rainy season was 0.3, and 0.2 during the dry season (satellite-derived) (Table 4).

Measures of small-scale agriculture and nearby house density were heterogeneous along the urban–rural continuum in Blantyre, Malawi. Although the proportion of households producing small-scale agriculture tended to increase with distance from the city centre, there were households within Blantyre city limits engaged in small-scale crop production. Likewise, there were clusters within Blantyre city limits with low nearby-house density and clusters outside of Blantyre city limits situated in high house density (Fig. 3a, b).

### Bivariate analysis

Negative binomial regression models were used to quantify the associations of explanatory variables with the number of female *Anopheles* mosquitoes in each household, using separate analyses for *An. funestus* and *An. arabiensis*.

Single statistically significant predictors of greater household-level abundances of both *An. funestus* and *An. arabiensis* included more children under-5 years old, use of a bed net the preceding night, greater distance from the city centre, survey during the rainy season, higher proportion of surrounding land used for cropping (satellite-derived), presence of small-scale agriculture within a 50 m radius around household (direct observation), an NDVI during the dry season of > 0.1 and ≤ 0.4, typically corresponding to shrub/grassland (satellite-derived), ownership of goats, and having a thatched roof (vs. iron sheets) (Table 3). It is of note that similar associations were observed between *Anopheles* spp. abundances and measures of small-scale agriculture originating from various sources, including direct observation and satellite-derived measures of NDVI and percentage of land used for cropping.

**Table 3:** Bivariate negative binomial models of association between species-specific mosquito abundances and various predictors

	*An. arabiensis*		*An. funestus*	
	95% CI	*P* value	95% CI	P value
Demographics				
Number of rooms	0.8 (0.7, 0.9)	< *0.01*	0.7 (0.6, 0.8)	*0.0001*
Number of children under age 5	1.9 (1.3, 2.7)	< *0.001*	1.9 (1.3, 2.8)	< *0.001*
Number slept in the house	1.0 (0.8, 1.1)	0.75	0.9 (0.8, 1.1)	0.42
Age of household head (years)	0.99 (0.98, 1.0)	0.09	0.98 (0.97, 0.99)	< *0.01*
Male head of household	1.1 (0.7, 1.7)	0.78	1.3 (0.8, 2.0)	0.23
Highest educational attainment of head of household		< *0.001*		*0.0001*
No formal education	Ref		Ref	
Some primary education	1.3 (0.5, 3.2)		2.8 (1.2, 6.9)	
Some secondary education	0.5 (0.2, 1.3)		1.6 (0.6, 4.0)	
Some college	0.4 (0.1, 1.2)		0.2 (0.1, 0.6)	
Malaria prevention practices				
Total number of nets per household	0.8 (0.6, 0.9)	*0.01*	0.7 (0.6, 0.9)	< *0.001*
Respondent used a net night prior to study	2.5 (1.5, 3.9)	*0.0001*	2.2 (1.4, 3.4)	< *0.001*
Other family members used a net night prior to study	3.2 (2.0, 4.9)	*0.0001*	2.5 (1.6, 3.9)	*0.0001*
Ave. number of nets per person	0.2 (0.1, 0.5)	*0.0001*	0.3 (0.2, 0.6)	< *0.001*
Household environmental characteristics (50 m buffer)				
Within Blantyre city limits	0.1 (0.1, 0.2)	*0.0001*	0.02 (0.01, 0.03)	*0.0001*
Increasing distance from city centre (2.5 km intervals)	1.5 (1.4, 1.7)	*0.0001*	1.7 (1.5, 1.9)	*0.0001*
Section/region				
Lunzu (1)	Ref	Ref	Ref	Ref
Chileka (2)	1.1 (0.5, 2.6)	0.75	1.8 (0.9, 3.6)	0.08
Chilomoni (3)	1.0 (0.4, 2.4)	0.97	1.1 (0.5, 2.1)	0.87
Mpemba (4)	0.1 (0, 0.3)	*0.0001*	0.1 (0, 0.1)	*0.0001*
Chigumula (5)	0.2 (0.1, 0.4)	*0.0001*	0.1 (0, 0.1)	*0.0001*
Mikolongwe (6)	0.9 (0.4, 2.0)	0.75	0.1 (0.1, 0.2)	*0.0001*
Kachere (7)	0.3 (0.1, 0.7)	< *0.01*	0 (0, 0.1)	*0.0001*
Machinjiri (8)	0.5 (0.2, 1.2)	0.11	0.1 (0, 0.2)	*0.0001*
Rainy (vs. dry) season	46.1 (29.6, 71.6)	*0.0001*	15.3 (10.5, 22.4)	*0.0001*
Elevation (m)	0.996 (0.995, 0.997)	*0.0001*	0.992 (0.991, 0.993)	*0.0001*
Number nearby households	0.9 (0.9, 1.0)	*0.0001*	0.9 (0.8, 0.9)	*0.0001*
Amount of land used for growing crops (%)	2.3 (1.2, 4.2)	< *0.01*	1.8 (1.0, 3.1)	*0.04*
NDVI (rainy season)	0.4 (0, 6.5)	0.50	2.6 (0.2, 30.4)	0.44
NDVI category (rainy season)				
≤ 0.1 (barren: rock/sand/urban)	Ref	Ref	Ref	Ref
> 0.1 and ≤ 0.4 (shrub/grassland)	2.6 (0.1, 94.7)	0.60	0.6 (0, 17.6)	0.80
> 0.4 and ≤ 1 (temperate/tropical rainforest)	1.8 (0, 69.9)	0.74	0.8 (0, 22.3)	0.89
NDVI (dry season)	96.5 (0, 212,362.4)	0.25	0.1 (0, 304.7)	0.53
NDVI category (dry season)				
≤ 0.1 (barren: rock/sand/urban)	Ref	Ref	Ref	Ref
> 0.1 and ≤ 0.4 (shrub/grassland)	19.4 (5.2, 72.1)	< *.0001*	111.6 (22, 566.7)	< *.0001*
> 0.4 and ≤ 1 (temperate/tropical rainforest)	NA	NA	NA	NA
Agriculture	5.8 (3.8, 8.8)	*0.0001*	4.0 (2.6, 6.1)	*0.0001*
Fruit trees	0.9 (0.6, 1.6)	0.82	0.8 (0.5, 1.2)	0.27
Grazing	0.6 (0.3, 1.0)	0.06	0.5 (0.3, 0.9)	*0.02*
Forest	0.4 (0.2, 1.1)	0.08	0.2 (0.1, 0.4)	*0.0001*
Ownership of goats	3.1 (1.7, 5.8)	< *0.001*	3.9 (2.1, 7.0)	*0.0001*
Ownership of chickens	1.3 (0.8, 2.1)	0.25	1.7 (1.1, 2.6)	*0.03*
Housing construction				
Closed (vs. fully/partially open) windows	0.9 (0.4, 1.8)	0.72	0.5 (0.2, 0.9)	*0.03*
Closed (vs. fully/partially open) eaves	2.0 (1.0, 4.3)	0.06	2.2 (1.1, 4.5)	*0.03*
Roof type				
Iron sheets	Ref	Ref	Ref	Ref
Thatched	3.0 (1.8, 4.9)	*0.0001*	4.1 (2.5, 6.6)	*0.0001*
Tile	0.5 (0, 37.0)	0.74	0.6 (0, 31.2)	0.80

Italic values indicate significance of p value (*p* < 0.05)

Fewer mosquitoes of both species were independently associated with more rooms in the house, higher educational attainment of the household head, greater total number of bed nets, greater average number of bed nets per person, location within Blantyre city limits, location within certain sections of the study area (Mpemba, Chigumula, and Kachere), and greater nearby house density (Table 3).

Greater abundances of *An. funestus* alone were associated with owning chickens and having closed (vs. open or partially open) eaves. On the other hand, fewer *An. funestus* were associated with certain sections of the study area (Mikolongwe and Machinjiri), presence of animals for nearby grazing, location near forested areas, and having closed (vs. open or partially open) windows (Table 3). These findings may be suggestive of differences in vector behaviour.

To consider potential confounding, variables that were significantly associated with both *An. funestus* and *An. arabiensis* were further evaluated for significant relationships with the presence of small-scale agriculture. Potential confounders were determined to be the number of under 5-year-olds, educational attainment of the head of household, total number of bed nets per household, the household-average of bed nets, location within Blantyre city limits, increasing distance from city centre, rainy season, nearby house density, percentage of cropped land within a 50 m radius of the household, NDVI category during the dry season, and ownership of goats (Tables 3 and 4). Not all variables identified as potential confounders were included in the final models, as percent crop and NDVI category during the dry season were both highly correlated with the main predictor of interest, small-scale agriculture.

**Table 4:** Bivariate analysis of household demographics, behavioural factors, and peri-domestic environmental characteristics by presence/absence of agriculture

	All households			Agriculture absent			Agriculture present			P value (T test or Chi square)
	N	Mean or Freq	SD or %	N	Mean or Freq	SD or %	N	Mean or Freq	SD or %	
Demographics										
Number of rooms	1546	3.7	1.9	726	3.8	2.1	820	3.6	1.6	0.14
Number of children under age 5	1520	0.6	0.8	700	0.5	0.7	820	0.6	0.8	< *0.01*
Number slept in the house	1520	3.8	1.9	700	3.7	2.0	820	3.9	1.7	0.13
Age of household head (years)	1520	40.5	16.8	700	40.7	16.4	820	40.3	17.1	0.64
Male head of household	1520	621	40.9%	700	290	41.4%	820	331	40.4%	0.67
Highest educational attainment of head of household	1509			692			817			< *.0001*
No formal education		95	6.3%		35	5.1%		60	7.3%	
Some primary education		792	52.5%		315	45.5%		477	58.4%	
Some secondary education		487	32.3%		250	36.1%		237	29.0%	
Some college		135	8.9%		92	13.3%		43	5.3%	
Malaria prevention practices										
Total number of nets per household	1546	1.6	1.3	726	1.7	1.4	820	1.5	1.2	*0.0001*
Respondent used a net night prior to study	1548	986	63.7%	728	463	63.6%	820	523	63.8%	0.94
Other family members used a net night prior to study	1548	917	59.2%	728	414	56.9%	820	503	61.3%	0.07
Ave. number of nets per person	1520	0.5	0.4	700	0.5	0.4	820	0.4	0.4	< *.0001*
Household environmental characteristics (50 m buffer)										
Within Blantyre city limits	1548	532	34.4%	728	352	48.4%	820	180	22.0%	< *.0001*
Increasing distance from city centre (2.5 km intervals)	1548			728			820			< *.0001*
2.5 km		190	12.3%		143	19.6%		47	5.7%	
5 km		193	12.5%		132	18.1%		61	7.4%	
7.5 km		196	12.7%		115	15.8%		81	9.9%	
10 km		193	12.5%		82	11.3%		111	13.5%	
12.5 km		193	12.5%		81	11.1%		112	13.7%	
15 km		196	12.7%		61	8.4%		135	16.5%	
17.5 km		194	12.5%		51	7.0%		143	17.4%	
20 km		193	12.5%		63	8.7%		130	15.9%	
Section/region	1548			728			820			< *.0001*
Lunzu (1)		190	12.3%		113	15.5%		74	9.0%	
Chileka (2)		193	12.5%		106	14.6%		84	10.2%	
Chilomoni (3)		196	12.7%		86	11.8%		104	12.7%	
Mpemba (4)		193	12.5%		120	16.5%		74	9.0%	
Chigumula (5)		193	12.5%		77	10.6%		122	14.9%	
Mikolongwe (6)		196	12.7%		82	11.3%		113	13.8%	
Kachere (7)		194	12.5%		84	11.5%		115	14.0%	
Machinjiri (8)		193	12.5%		60	8.2%		134	16.3%	
Rainy season (vs. dry)	1548	618	39.9%	728	124	17.0%	820	494	60.2%	< *.0001*
Elevation (m)	1548	945.0	175.9	728	947.2	165.3	820	943.0	184.9	0.64
Number nearby households	1548	8.3	10.4	728	9.6	13.0	820	7.2	7.2	< *.0001*
Amount of land used for growing crops (%)	1548	25.8	40.4	728	21.4	38.7	820	29.6	41.5	< *.0001*
NDVI (rainy season)	1548	0.3	0.1	728	0.3	0.1	820	0.3	0.1	< *.0001*
NDVI category (rainy season)	1548			728			820			0.20
≤ 0.1 (barren: rock/sand/urban)		4	0.3%		2	0.3%		6	0.7%	
> 0.1 and ≤ 0.4 (shrub/grassland)		1291	83.4%		617	84.8%		674	82.2%	
> 0.4 and ≤ 1 (temperate/tropical rainforest)		251	16.2%		107	14.7%		144	17.6%	
NDVI (dry season)	1548	0.2	0.0	728	0.2	0.0	820	0.2	0.0	< *.0001*
NDVI category (dry season)	1548			728			820			< *.0001*
≤ 0.1 (barren: rock/sand/urban)		92	5.9%		73	10.0%		19	2.3%	
> 0.1 and ≤ 0.4 (shrub/grassland)		1456	94.1%		655	90.0%		801	97.7%	
> 0.4 and ≤ 1 (temperate/tropical rainforest)		0	0%		0	0%		0	0%	
Fruit trees	1548	1134	73.3%	728	439	60.3%	820	695	84.8%	< *.0001*
Grazing	1548	275	17.8%	728	217	8.7%	820	212	25.9%	< *.0001*
Forest	1548	108	7.0%	728	65	8.9%	820	43	5.2%	< *0.01*
Ownership of goats	1546	221	14.3%	726	76	10.5%	820	145	17.7%	< *.0001*
Ownership of chickens	1546	582	37.6%	726	248	34.2%	820	334	40.7%	*0.01*
Housing construction										
Closed (vs. fully/partially open) windows	1527	175	11.5%	719	93	12.9%	808	82	10.2%	0.09
Closed (vs. fully/partially open) eaves	1537	146	9.5%	720	76	10.6%	817	70	8.6%	0.19
Roof type	1512			694			818			< *.0001*
Iron sheets		1129	74.7%		565	81.4%		564	68.9%	
Thatched		379	25.1%		126	18.2%		253	30.9%	
Tile		4	0.3%		3	0.4%		1	0.1%	

Italic values indicate significance of p value (*p* < 0.05)

### Multivariate analysis

Multivariate negative binomial models were used to quantify the association of small-scale agriculture and various urbanity measures with the number of female *Anopheles* mosquitoes in each household, adjusting for confounding. *Anopheles funestus* and *An. arabiensis* were analysed separately for a total of 1387 household-visits after excluding those with missing outcome, exposure, or risk factor information.

Small-scale agriculture and increasing distance from city centre (in 2.5 km intervals) were significantly associated with increased abundances of *An. funestus* and *An. arabiensis*, while location within Blantyre city limits and greater nearby house density were significantly associated with decreased abundances of both *Anopheles* species (Table 5). These relationships remained similar after adjusting for the number of bed nets per person, number of children under 5 years old, education level, and rainy/dry season; however, the effect size of small-scale agriculture on *Anopheles* spp. abundances generally decreased after adjustment becoming non-significant (Table 6). As expected, season was a strong predictor of *Anopheles* abundances; inclusion of rainy/dry season in the models attenuated the effect of agriculture on *An. arabiensis* and reversed the direction of the association between agriculture and *An. funestus.* The effects of various urbanicity measures on *Anopheles* spp. abundances remained stable and significant after adjusting for confounding.

**Table 5:** Unadjusted multivariate negative binomial models of associations between *Anopheles* abundances, presence of small-scale agriculture, and urbanicity measures

	*An. arabiensis*		*An. funestus*	
	95% CI	P value	95% CI	P value
Model 1a				
Agriculture	5.2 (3.4, 7.9)	< *.0001*	2.7 (1.8, 4.0)	< *.0001*
Within city limits	0.2 (0.1, 0.3)	< *.0001*	0.02 (0.01, 0.04)	< *.0001*
Model 2a				
Agriculture	5.6 (3.7, 8.6)	< *.0001*	3.4 (2.3, 5.1)	< *.0001*
Increasing distance	1.4 (1.3, 1.6)	< *.0001*	1.6 (1.4, 1.7)	< *.0001*
Model 3a				
Agriculture	5.5 (3.6, 8.4)	< *.0001*	3.7 (2.4, 5.6)	< *.0001*
House density^a^	0.5 (0.4, 0.8)	*0.001*	0.3 (0.2, 0.4)	< *.0001*

Italic values indicate significance of p value (*p* < 0.05)

^a^Units are an additional 10 households within a 50 m radius of the sampled household

**Table 6:** Multivariate negative binomial models of associations between *Anopheles* abundances, presence of small-scale agriculture, and urbanicity measures, adjusted for number of nets per person, number of children under 5 years old, education level, and rainy season

	*An. arabiensis*		*An. funestus*	
	95% CI	P value	95% CI	P value
Model 1b				
Agriculture	1.4 (0.8, 2.2)	0.21	0.6 (0.4, 0.9)	*0.01*
Within city limits	0.2 (0.1, 0.2)	< *.0001*	0.02 (0.01, 0.04)	< *.0001*
Model 2b				
Agriculture	1.6 (1.0, 2.5)	0.07	0.7 (0.4, 1.2)	0.19
Increasing distance	1.3 (1.2, 1.5)	< *.0001*	1.4 (1.3, 1.6)	< *.0001*
Model 3b				
Agriculture	1.5 (1.0, 2.5)	0.08	0.7 (0.5, 1.2)	0.21
House density^a^	0.5 (0.4, 0.7)	< *0.001*	0.2 (0.1, 0.3)	< *.0001*

Italic values indicate significance of p value (*p* < 0.05)

^a^Units are an additional 10 households within a 50 m radius of the sampled household

Interactions were assessed between the main effect, presence of small-scale agriculture, and various urbanicity measures. A significant positive interaction was observed between agriculture and “urban” (within Blantyre city limits), while a significant negative interaction was observed between agriculture and increasing distance from city centre (increasingly rural) for *An. arabiensis* only (Table 7). These findings imply that the presence of small-scale agriculture is more predictive of *An. arabiensis* abundance at houses within Blantyre city limits and for houses increasingly close to Blantyre city centre. There was no significant interaction found between small-scale agriculture and nearby house density for either *An. arabiensis* or *An. funestus*.

**Table 7:** Multivariate negative binomial models of associations and interactions between *Anopheles* abundances, presence of small-scale agriculture, and urbanicity measures

	*An. arabiensis*		*An. funestus*	
	95% CI	P value	95% CI	P value
Model 1c				
Agriculture	3.8 (2.3, 6.2)	< *.0001*	2.7 (1.7, 4.2)	< *.0001*
Within city limits	0.1 (0, 0.2)	< *.0001*	0.02 (0.01, 0.05)	< *.0001*
Agriculture^a^ within city limits	3.4 (1.2, 9.6)	*0.02*	1.0 (0.4, 2.8)	1.00
Model 2c				
Agriculture	15.6 (5.4, 45.2)	< *.0001*	8.2 (2.9, 23.8)	< *.0001*
Increasing distance	1.6 (1.4, 1.8)	< *.0001*	1.7 (1.5, 2.0)	< *.0001*
Agriculture^a^ increasing distance	0.8 (0.7, 1.0)	*0.04*	0.8 (0.7, 1.0)	0.07
Model 3c				
Agriculture	5.3 (2.7, 10.5)	<*.0001*	4.2 (2.0, 8.9)	*0.0001*
Housing density	0.5 (0.3, 0.9)	*0.02*	0.3 (0.2, 0.6)	< *0.001*
Agriculture^a^ house density^a^	1.0 (0.5, 2.2)	0.91	0.8 (0.3, 2.0)	0.65

Italic values indicate significance of p value (*p* < 0.05)

^a^Units are an additional 10 households within a 50 m radius of the sampled household

## Discussion

The reasons why malaria persists in many urbanizing areas of sub-Saharan Africa are multifaceted and not well understood. One key question is whether incident cases in urban settings are resulting from transmission there, or from infection acquired during travel to more rural settings, which then is transported back to urban residences. Understanding such drivers of malaria risk is critical in contexts experiencing rapid urbanization, such as Malawi, where the urban growth rate is 4% per annum [4]. Malaria prevention among the ~ 3.2 million (17%) of Malawi’s population living in an urban setting is limited by inadequate knowledge of what determines risk [4]. One challenge is the diverse and imprecise definitions of “urban” or “rural”, which may be misleading and can obscure local heterogeneity across the risk landscape. To understand what constitutes risk may be further complicated by uneven urbanization, making it difficult to prioritize where resources and interventions should be directed. Results from this study demonstrate that small-scale crop production and other peri-domestic environmental factors are major influences on the local abundance of malaria vectors, even in high-density urban areas.

While *An. funestus* and *An. arabiensis* were often associated with similar risk factors, several species-specific risk factors were also identified, implying that different strategies may need to be utilized to address species-specific malaria risk. Greater *An. funestus* and *An. arabiensis* abundances inside households were predicted by the presence of more under 5-year-olds, greater distance from the city centre, rainy season, more peri-domestic land used for crop production, an NDVI during the dry season of > 0.1 and ≤ 0.4, typically corresponding to shrub/grassland, but which could also reflect the presence of small-scale agriculture in this setting, goat ownership, and having a thatched vs. iron or tile roof. These associations are generally consistent with what has been seen in other similar SSA high-transmission settings and have been explained by various biological and behavioural pathways [35–38]. More *An. funestus* alone were predicted by chicken ownership and having closed (vs. open or partially open) eaves, suggestive of differences in species behaviour and species-specific risk.

Fewer mosquitoes of both species were independently predicted in households with more rooms, a higher level of educational attainment of the household head, location within Blantyre city limits, location in certain sections of the study area, and higher nearby house density. Plausible mechanisms for these associations have also been proposed in other studies, and mostly involve physical or knowledge-based relationships to mosquito breeding or household access [36, 37]. Fewer *An. funestus* only were associated with other sections of the study area, presence of animals for nearby grazing, location near forested areas, and having closed (vs. open or partially open) windows [39].

The number of bed nets per household and their reported use were associated differently with vector abundance. More *An. funestus* and *An. arabiensis* were observed in households with greater bed net use the night preceding the survey; however, fewer mosquitoes of both species were observed in households where more total bed nets were present, and with a higher number of bed nets per person. These findings are provocative and suggest that people more readily use nets when mosquitoes are more obvious or annoying, while the presence of more nets inside the dwelling, regardless of their nighttime use, may reduce survival or repel mosquitoes from these indoor settings. McCann et al. found that indoor *Anopheles* density decreased with increasing LLIN use when analysed categorically, which is contrary to the findings from this study [40]. One possible explanation is the cross-sectional nature of our study design. In areas where mosquitoes are more abundant, people may tend to use mosquito nets more frequently or consistently; however, it is not possible to definitely assess the direction of the association from this study alone.

As expected, the effect of seasonality on *Anopheles* abundances was large and significant, and impacted the other observed effects. Season attenuated the model effect size of small-scale agriculture on *An. arabiensis* and reversed the direction of the association between small-scale agriculture and *An. funestus*. Seasonality is a well-known predictor of *Plasmodium* transmission, as heavy rains in these settings can often allow for *Anopheles* breeding habitats to expand [35].

Finally, small-scale subsistence agriculture was found to be associated with greater *Anopheles* spp. abundance in urban and peri-urban Blantyre, even after adjusting for degree of urbanicity and other confounders. This suggests that small-scale agriculture is an important risk factor for greater malaria vector abundance, even in urbanized areas. Furthermore, results demonstrated that small-scale agriculture was more important to *Anopheles* spp. abundance in “urban” households located within city limits, as evidenced by significant interaction terms between small-scale agriculture and urbanicity measures. In other words, small-scale agriculture is more predictive of *Anopheles* spp. presence in households located within city limits and at distances closer to the city centre, but less predicative of *Anopheles* spp. presence in households located outside of Blantyre city limits and at distances further from the city centre. This observation implies there are additional factors at play in more rural area households which were not adequately captured in this study.

## Conclusion

The role of environmental characteristics, particularly small-scale agriculture, in the reproduction and survival of malaria vectors in urban habitats is critical, yet still enigmatic. Findings from this study indicate that poverty, poor quality housing, and small-scale agriculture in urban settings contribute to conditions that amplify anopheline mosquito abundance, particularly for adaptable species such as *An. funestus*, and may thereby augment the risk of urban transmission. Household-level and peri-domestic environmental characteristics found to be associated with malaria vector abundance were identified by characterizing the presence of *Anopheles* species along an urban–rural continuum in this highly endemic transmission setting. These insights contribute to a better understanding of heterogeneous risk along the urban–rural continuum that impact on local *Plasmodium* transmission, and that require elucidation for malaria-prevention efforts to become more effective.

## Additional file

**Additional file 1.** Details of laboratory methods for mosquito speciation.

## Abbreviations

AIC: Akaike information criterion
CDC: Centers for Disease Control and Prevention
DEM: digital elevation model
DNA: deoxyribonucleic acid
GIS: geographic information systems
GPS: global positioning system
ICEMR: International Center of Excellence for Malaria Research
IRB: Institutional Review Board
LLIN: long-lasting insecticidal net
NIR: near infrared
NDVI: normalized difference vegetation index
PCR: polymerase chain reaction
SAS: Statistical Analysis System
SSA: Sub-Saharan Africa
WHO: World Health Organization
