# Supplementary material for: Household-level and surrounding peri-domestic environmental characteristics associated with malaria vectors Anopheles arabiensis and Anopheles funestus along an urban–rural continuum in Blantyre, Malawi
Source: Malar J. 2018 Jun 8;17:229. doi: 10.1186/s12936-018-2375-5 (PMC5994107; doi:10.1186/s12936-018-2375-5)
Supplement: Supplementary file 1 — Additional file 1. Details of laboratory methods for mosquito speciation. [file 12936_2018_2375_MOESM1_ESM.docx]

**Supplemental Information**

***Mosquito Speciation***

DNA from abdomens was extracted using the Potassium Acetate technique. Briefly samples were ground in 100 µl of a 4:1 mixture of homogenization buffer (0.1M NaCl, 0.2M Sucrose, 0.01M EDTA, 0.03 M Tris Base, pH=8.0) and Lysis Buffer (0.25M EDTA, 2.5% SDS, 0.5M Tris Base, pH=9.2). Samples were incubated at 65˚C for 30 minutes. Subsequently, 14 µl of a 3M Potassium Acetate solution was added and samples were incubated on ice for 30 minutes. Samples were spun at 16,800g for 10 minutes. Supernatant was transferred to a new tube, 200 µl of 100% Ethanol was added, and samples were placed at -20˚C for 20 minutes. Samples were again spun at 16,800g for 20 minutes. Supernatant was then discarded, and the pellet was washed first in 70% ethanol and then in 100% ethanol. The final pellet was dried overnight and re-suspended in 200 µl TE buffer.

DNA was subjected to PCR amplification. Briefly, either 2 µl of DNA (*Anopheles* *funestus*) or 1 µl (*Anopheles* *gambiae*) was subjected to polymerase chain reaction for 40 cycles using primers based on either species-specific single nucleotide polymorphisms in the internal transcribed spacer region or the intergenic spacer region. For *Anopheles* *funestus* amplification a universal forward primer (5’TGTGAACTGCAGGACACAT3’) was matched with specific reverse primers for *Anopheles vaneedeni* (5’TGTCGACTTGGTAGCCGAAC3’), *Anopheles funestus* (5’GCATCGATGGGTTAATCATG3’), and *Anopheles parensis* (5’TGCGGTCCCAAGCTAGGTTC3’). For *gambiae* complex a universal forward primer (5’GTGGGCCCCTTCCTCGATGT3’) was matched with a specific reverse primer: *Anopheles arabiensis* (5’AAGTGTCCTTCTCCATCCTA3’) or *Anopheles gambiae ss* (5’CTGGTTTGGTCGGCACGTTT3’). PCR product was run on 2% agarose gels and compared to standards for each species that were amplified in parallel.
